# Very Preterm Children Gut Microbiota Comparison at the Neonatal Period of 1 Month and 3.5 Years of Life

**DOI:** 10.3389/fmicb.2022.919317

**Published:** 2022-07-22

**Authors:** Gaël Toubon, Marie-José Butel, Jean-Christophe Rozé, Patricia Lepage, Johanne Delannoy, Pierre-Yves Ancel, Marie-Aline Charles, Julio Aires, for the EPIFLORE Study Group, Clotilde Rousseau

**Affiliations:** (Federation of University Hospital, PREMA, UMR-S 1139, Faculty of Pharmacy, INSERM and Paris Descartes University); (INRA, UMR 1319 MICALIS); J-CR (Department of Neonatal Medicine, Nantes University Hospital); (INSERM, U1153, Obstetrical, Perinatal and Pediatric Epidemiology Team, Epidemiology and Biostatistics Sorbonne); (Department of Neonatal Medicine, Assistance Publique Hopitaux de Paris, Necker Enfants Malades Hospital); (Department of Neonatal Medicine, Hopital de la Croix-Rousse, Hospices Civils de Lyon); (Department of Neonatology, Faculte de Medecine, Aix-Marseille Université); (Division of Neonatology, Department of Perinatology, Armand Trousseau Hospital); (Department of Neonatalogy, Université Paris 7, Robert-Debre Hospital, Assistance Publique Hopitaux de Paris); (Department of Neonatal Medicine, Lille University Hospital); (Mothers and Children Hospital, Hospices Civils de Lyon); (Department of Neonatal Medicine, Montpellier University Hospital); (Department of Neonatal Medicine, Nantes University Hospital); (Department of Neonatal Medicine, Rennes University Hospital); (Department of Neonatal Medicine, Toulouse University Hospital); (Department of Neonatalogy, Tours University Hospital); (Department of Neonatal Medicine, Nancy University Hospital); (Department of Neonatal Medicine, Angers University Hospital); (Department of Pediatrics, Besancon University Hospital); (Department of Neonatal Medicine, Louis Mourier Hospital, Assistance Publique Hopitaux de Paris); (Department of Neonatal Medicine, Centre Hospitalier Intercommunal); (Department of Neonatal Medicine, Cochin University Hospital); ^1^INSERM, UMR1153 Centre de Recherche Épidémiologie et Statistiques (CRESS), Université Paris Cité, Paris, France; ^2^Université Paris Cité, INSERM, UMR-S 1139, Physiopathologie et Pharmacotoxicologie Placentaire Humaine Microbiote Pré & Postnatal (3PHM), Paris, France; ^3^FHU PREMA, Fighting Prematurity, Paris, France; ^4^INRAE, UMR 1280, Physiologie des Adaptations Nutritionnelles (PhAN), Université hospitalière de Nantes, Nantes, France; ^5^INRAE, UMR 1319, AgrosParisTech, Institut Micalis, Université Paris-Saclay, Paris, France

**Keywords:** prematurity, gut microbiota, DOHaD, children, enterotypes

## Abstract

Prematurity is a risk factor for dysbiosis of the gut microbiota due to particular birth conditions and frequent prolonged hospitalization of neonates. Although gut microbiota colonization after birth and its establishment during the hospitalization period have been studied in preterm infants, data on gut microbiota following discharge, particularly during early childhood, are scarce. The present study investigated the relationship between gut microbiota at 1 month after birth (hospitalization period) and 3.5 years of age in 159 preterm children belonging to the French EPIFLORE prospective observational cohort study. Analysis using bacterial 16S rRNA gene sequencing showed that the gut microbiota of preterm neonates at 1 month was highly variable and characterized by six distinct enterotypes. In contrast, the gut microbiota of the same children at 3.5 years of age showed less variability, with only two discrete enterotypes. An absence of association between enterotypes at 1 month and 3.5 years of age was observed. While the alpha diversity of gut microbiota significantly increased between 1 month and 3.5 years of age, for both alpha and beta diversities, there was no correlation between the 1-month and 3.5-years time points. Comparison at 3.5 years between children born either preterm (*n* = 159) or full-term (*n* = 200) showed no differences in terms of enterotypes, but preterm children harbored a lower Shannon diversity index and a different overall composition of microbiota than full-term children. This study suggests that the characteristics of the early gut microbiota of preterm children are not predictive of the microbial community composition at 3.5 years of age. However, the impact of gestational age is still noticeable on the gut microbiota up to 3.5 years of age.

## Introduction

The human microbiota has become a research topic of utmost interest in recent years, with several studies highlighting its potential impact on physiology and, as a result, its possible effects on health and disease (Fan and Pedersen, 2021; Sarkar et al., 2021). Recently, substantial efforts have focused on studying infant gut microbiota with the emergence of the concept of Developmental Origins of Health and Disease (DOHaD) (Butel et al., 2018) and the critical first 1.000 days of life window hypothesis (Wopereis et al., 2014; Robertson et al., 2019). Prematurity, accounting for approximately 10% of births worldwide (Blencowe et al., 2012; Chawanpaiboon et al., 2019), is a risk factor for early gut dysbiosis, which is suggested to be associated with necrotizing enterocolitis (Rozé et al., 2017), growth delay (Tirone et al., 2019), cognitive impairment (Rozé et al., 2020), and brain damage (Seki et al., 2021). In very preterm neonates, gut microbiota is characterized by high inter-individual variability (Yee et al., 2019; Rozé et al., 2020) that can be attributed to varying birth conditions and extended exposure to the neonatal intensive care unit (NICU) environment and practices. Indeed, preterm infants are more often born *via* cesarean section and are frequently administered broad-spectrum antibiotics. Furthermore, gestational age influences the dynamics of the intestinal bacterial establishment (La Rosa et al., 2014). The early establishment of preterm gut microbiota is characterized by a dominance of opportunistic and potentially pathogenic bacteria, such as *Enterobacter*, *Enterococcus*, and *Staphylococcus*, and a delay in colonization by *Bifidobacterium* and *Bacteroides* (Arboleya et al., 2012; Barrett et al., 2013; La Rosa et al., 2014; Hill et al., 2017; Korpela et al., 2018). Several studies have highlighted the influence of the NICU environment on the establishment of preterm neonate gut microbiota (Hartz et al., 2015; Moles et al., 2015; D’Agata et al., 2019; Tauchi et al., 2019; Yap et al., 2021), notably during hospitalization (Rozé et al., 2020), and the existence of overlap of microbial taxa between NICU surfaces and the gut of preterm neonates (Brooks et al., 2014). Thus, while the infant gut microbiota establishment during the first weeks and months of life is documented, only a few studies have reported on the late development of gut microbiota in preterm children. Additionally, the relationship between gut microbiota and early-life neonatal factors, such as birth mode, breastfeeding, and antibiotic treatments after NICU discharge and during early childhood of preterm children, has been rarely studied, although it has been emphasized as a research priority (Groer et al., 2014). Indeed, to date, only a few studies with small cohorts have explored the development of gut microbiota in preterm children after 1 year of age (Gómez et al., 2017; Fouhy et al., 2019; Yee et al., 2019). These studies report an increase in alpha diversity over time and lower inter-individual variability in the gut microbiota between NICU discharge and early childhood. Furthermore, it has been suggested that gestational age at birth results in distinct microbial profiles extending up to 4 years of age (Fouhy et al., 2019). To the best of our knowledge, these data constitute the only available longitudinal evidence describing the gut microbiota of preterm children during early childhood. Therefore, given the paucity of data regarding this subject, there is a need to add substantial evidence related to prematurity and gut microbiota during early childhood, particularly in large cohorts.

This study aims to compare and characterize the gut microbiota of 159 preterm children from the EPIPAGE2 French nationwide cohort at two time points: at 1 month of life (NICU hospitalization) and at 3.5 years of age, when the child’s gut microbiota supposedly stabilizes toward a mature adult-like gut microbiota (Stewart et al., 2018). Additionally, we investigated factors previously reported to influence early gut microbiota development, such as gestational age (Groer et al., 2014; Gritz and Bhandari, 2015; Chernikova et al., 2018), birth mode (Dominguez-Bello et al., 2010; Portela et al., 2015; Stewart et al., 2018), birth weight (Groer et al., 2014; Unger et al., 2015), sex (Martin et al., 2016), antibiotics (Gibson et al., 2015; Tamburini et al., 2016), breastfeeding (Portela et al., 2015; Lemas et al., 2016; Pannaraj et al., 2017; Stewart et al., 2018), skin-to-skin contact (Hartz et al., 2015), and NICU strategies (Rozé et al., 2020). This study provides informative data concerning prematurity and the gut microbiota in early childhood based on a large and well-documented French multicentric cohort of preterm children.

## Materials and Methods

### EPIFLORE Study and ELFE Cohort

The preterm children included in this study were part of the EPIFLORE study (Ancel and Goffinet, 2014), an ancillary study of the EPIPAGE2 French national birth cohort that was launched in 2011 and included preterm neonates born between 24 and 31 weeks of gestation (Lorthe et al., 2021). The aim of EPIFLORE was to investigate the development of the intestinal microbiota and its relationship with early-life factors that influence its establishment and subsequent health outcomes in early childhood among very preterm children (<32 weeks of gestational age). The EPIFLORE study included clinical data and stool samples collected from 24 French NICUs (Rozé et al., 2020). In the present study, full-term children who were born in 2011 from the ELFE French national birth cohort (Charles et al., 2020) were included as controls to investigate the differences between the gut microbiota of preterm and full-term children at 3.5 years of age. Full-term singletons born after 37 weeks of gestation with fecal samples available at 3.5 years of age and at least 2 years of subsequent follow-up were randomly selected from the ELFE cohort.

### Participants and Sample Collection

The EPIFLORE study included 729 neonates, and stool samples were collected from 574 preterm neonates during hospitalization at a median age of 23 days (interquartile range [IQR], 22–26 days), which is referred to as 1-month sampling. An additional stool sample was obtained for 208 preterm children at a median age of 42.6 months (IQR, 42.2–43.4 months), referred to as 3.5 years of age sampling. In the current study, 159 preterm children with fecal samples collected at both time points were included. Additionally, stool samples of 200 full-term children from the ELFE cohort were collected at 3.5 years of age (median age, 42.1 months; IQR, 41.4–43.6 months) during the same period and using the same methodology as for preterm children (Supplementary Figure 1).

### DNA Extraction, Sequencing, and Data Processing

The total fecal DNA was extracted according to the International Human Microbiome Standards operating procedure (Dore et al., 2015), as previously reported (Rozé et al., 2020). We included negative controls that went through the same process as samples, from DNA extraction to sequencing. Sequencing was performed on the GeT-PlaGe platform of Génopole (Toulouse Midi-Pyrénées, France) using Illumina Miseq technology (V3, 2 × 250 bp) targeting the V3-V4 primers (V3fwd: ACGGRAGGCAGCAG, V4rev: AGGATTAGATACCCTGGTA) regions of the 16S bacterial rRNA gene. Raw sequences were analyzed using the pipeline “Find Rapidly OTU with Galaxy Solution” (FROGS) version 3.2 (September 2021) from the Galaxy software framework (Escudié et al., 2018). Sequences were checked for quality using FastQC (threshold 20–38). After trimming barcodes, the sequences were filtered (length of the reads ranged from 435 to 459 bp) and clustered into operational taxonomic units (OTUs) using the swarm clustering method implemented in the FROGS version 3.2 pipeline. OTUs representing less than 0.005% (788,210 reads) of all the sequences, the majority corresponding to singleton OTUs, were discarded (Bokulich et al., 2013). Taxonomic affiliation was assigned to the OTUs using the Silva 138.1 pintail100 database (September 2021) yielding 99% of the sequences affiliated with ≥99% identity and 100% coverage. A total of 11,526,400 reads (median, 22,619 reads per sample) were obtained from 16S rRNA gene sequencing. To adjust for the influence of uneven sampling depth, each sample was rarefied to the minimum sampling depth of the dataset (5,201 reads, Supplementary Figure 2), and rarefied data were used for all downstream analyses unless stated otherwise.

### Microbiota Analysis: Diversity and Composition

The Chao1 richness estimate (richness) and Shannon index (diversity) were calculated to investigate the patterns of microbial community diversity in the gut microbiota of preterm and full-term children. For beta diversity analysis, we computed dissimilarity matrices using Bray–Curtis and Unifrac distances. The Bray–Curtis dissimilarity distance reflects community composition considering the abundance of taxa, while the Unifrac distance considers the phylogenetic relationships among members of the bacterial communities. Gut microbiota inter-individual variability was visualized using principal coordinate analysis (PCoA/MDS) based on dissimilarity matrices. Stratification of the cohort based on gut microbial composition was performed according to the previously reported enterotyping guidelines (Arumugam et al., 2011). Briefly, the relative abundances of the classified genera were used to produce a Jensen–Shannon divergence (JSD) distance matrix between samples. Based on the obtained distance matrix, samples were clustered using Partitioning Around Medoids (PAM) algorithm. The optimal number of clusters was chosen by maximizing the Calinski–Harabasz index and was cross-validated with the silhouette index and prediction strength. The results of PAM clustering were visualized on PCoA biplots, and driver genera vectors were assessed using the “envfit” function. The vectors show the direction in the ordination space toward which genera change most rapidly and to which they have maximal correlations within the ordination configuration. The genus with the highest relative abundance in each group was considered the main contributor to each enterotype.

### Neonatal Characteristics and Neonatal Intensive Care Unit Strategies

In each NICU, data on children and mothers during the perinatal and neonatal periods were collected prospectively during hospitalization until discharge. Available clinical data included gestational age, birth mode, birth weight, sex, antibiotics, breastfeeding, skin-to-skin contact, and NICU strategies. The NICU strategies were previously characterized in the EPIPAGE2 study regarding early extubation or no intubation, use of sedation, direct breastfeeding, skin-to-skin practice, speed of progression of enteral feeding, and duration of primary and secondary antibiotic therapy (Rozé et al., 2020). All these practices concern the early period of hospitalization before the 1-month stool sampling period. The methods to characterize these strategies were applied as previously described (Rozé et al., 2020).

### Statistical Analysis

For the comparative analysis of alpha diversity and pairwise beta diversity between enterotypes and time points, Wilcoxon rank-sum tests and Kruskal–Wallis tests were performed. Hypothesis testing was carried out using permutational multivariate analysis of variance (PERMANOVA) based on 999 permutations to analyze the influence of neonatal factors on the gut microbiota and to compare the gut microbiota between preterm and full-term children. For all PERMANOVA analyses, Benjamini–Hochberg FDR (false discovery rate) correction was used to adjust for multiple testing. We implemented a differential abundance testing analysis in order to assess significant changes in abundance at the genus level between samples at 1 month and 3.5 years in the preterm population. As these methods can produce heterogeneous results (Nearing et al., 2021; Wallen, 2021), we used two different methods: Analysis of Compositions of Microbiomes with Bias Correction (ANCOM-BC) (Lin and Peddada, 2020) and ANOVA-Like Differential Expression tool for compositional data (ALDEx2) (Fernandes et al., 2014). Differences in abundance were based on non-rarefied counts for both ANCOM-BC and ALDex2, as both methods used their own normalization methods, and only genera with an abundance of 0.1 in at least 1% of the samples were used. We considered only concordant results between the two methods. *P*-values were corrected using the Benjamini–Hochberg method. Correlations of alpha- and beta-diversity community structures between 1 month and 3.5 years of age samples were assessed as follows: pairwise Spearman rank correlation test for alpha-diversity indexes; Mantel test for beta-diversity matrices using the Spearman rank correlation method based on 999 permutations. The association between enterotypes at both ages was tested using Fisher’s exact test. To analyze the association between microbiota enterotypes found at 3.5 years of age and both neonatal characteristics and NICU strategies (neonates hospitalized at day 7), we used the chi-squared tests for categorical variables and student tests for continuous variables in univariate analyses. Mixed-effects logistic regression with a random hospital intercept to consider the correlation between newborns in the same NICU was performed in multivariate analyses. All analyses were performed using the R software version 4.0.4 (R Foundation) and the packages phyloseq (v1.34.0), vegan (v2.6-0), cluster (v2.1.2), clusterSim (v0.49-2), fpc (v 2.2-9), ade4 (v1.7-16), ggplot2 (v 3.3.5), and lme4 (v1.1-27.1).

## Results

### Enrolled Children Population

Our analyzed sample of preterm children (*n* = 159) was compared with the total population enrolled in the EPIFLORE study (*n* = 570), without stool samples available at the two time points, that is, 1 month and 3.5 years of age (Supplementary Table 1). We found that the characteristics of our study population did not significantly differ from the total population enrolled in the EPIFLORE study except in terms of the mother’s age, the rate of mothers born outside France, and maternal level of education (Supplementary Table 1).

### Preterm Gut Microbiota at 1 Month of Age

**FIGURE 1:**
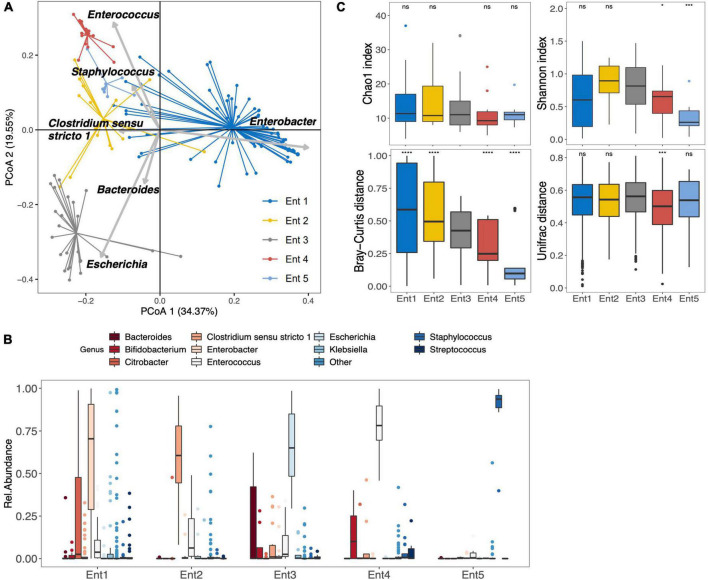
Gut microbiota composition and diversity of the 141 preterm infants at 1 month after birth. **(A)** Clustering based on the genus taxonomic profiles. Biplot arrows indicate the top six genera that drive the sample to different locations on the plot. **(B)** The boxplots represent the relative abundance of the top 10 genera distributed among the five enterotypes. The sixth enterotype is not represented because it is constituted by infants without sample amplification (low bacterial load). The boxplots show the smallest and largest values, 25 and 75% quartiles, the median, and outliers. **(C)** Boxplots of alpha diversity (top) assessed by Chao1 and Shannon index, and pairwise beta diversity dissimilarities (bottom) assessed by Bray–Curtis and Unifrac distances within enterotypes based on genus taxonomic profiles (*p < 0.05, ****p < 0.001, ns = *p* > 0.05, Reference group = Ent3); Ent, Enterotype.

Among the 159 stool samples analyzed at 1 month, 18 were non-amplifiable. The 141 neonates for whom sequencing data were obtained were partitioned into five bacterial enterotypes similar to those previously described by Rozé et al. (2020). In the present study, enterotypes 1–5 were characterized by a dominance of *Enterobacter* (*n* = 69), *Clostridium sensu stricto 1* (*n* = 18), *Escherichia* (*n* = 25), *Enterococcus* (*n* = 18), and *Staphylococcus* (*n* = 11), respectively (Figures 1A,B). At the OTU level, enterotypes 1–5 were characterized by the dominance of OTU_1_*E. hormaechei*, OTU_13_*C. neonatale*, OTU_2_*E. coli*, OTU_4_*E. faecalis*, and OTU_19_*S. caprae*, respectively (Supplementary Figure 3). Enterotype 2 (dominance of *Clostridium sensu stricto 1*) and enterotype 3 (dominance of *E. coli*) were associated with greater gestational age (Supplementary Figure 4). Enterotype 3 was considered to be the more mature microbiota and defined as the reference enterotype at 1 month, as previously reported (Rozé et al., 2020). Concerning the 18 non-amplifiable samples, they represented the enterotype 6 gathering infants with a low bacterial load (Rozé et al., 2020).

Community complexities of the five different enterotypes were compared at the genus level according to alpha (Chao 1 richness and Shannon index) and beta (Bray–Curtis and Unifrac distances) diversities (Figure 1C). Significant differences were observed in alpha diversity across all enterotypes (Shannon diversity index, *p* = 0.003). Compared to reference enterotype 3, enterotypes 4 and 5 showed a significantly lower alpha diversity (Shannon index, *p* < 0.05 and *p* < 0.001, respectively). However, pairwise beta-diversity comparisons between enterotypes showed significant overall differences across groups (Bray–Curtis and Unifrac distance dissimilarities, *p* < 10^–4^ for both). Higher distance dissimilarities among infants belonging to enterotypes 1 and 2 were observed when compared to the reference enterotype 3 (Bray–Curtis, *p* < 0.0001 for both enterotypes), suggesting higher inter-individual variation. Enterotypes 4 and 5 were characterized by lower pairwise distance dissimilarities within groups (Bray–Curtis, [*p* < 0.0001], Unifrac [*p* = 0.007] for Ent4, and Bray–Curtis [*p* < 0.0001] for Ent5). At the OTU level, significant differences were observed in alpha diversity across all enterotypes (Chao1 and Shannon diversity index, *p* = 0.005 and *p* = 0.008, respectively). Differences at the OTU level are further presented in the Supplementary Figure 3.

### Preterm Gut Microbiota at 3.5 Years of Age

**FIGURE 2:**
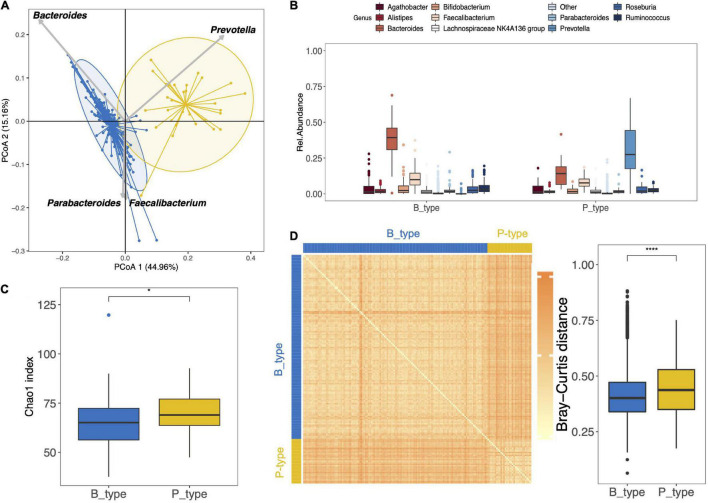
Gut microbiota composition and diversity of the 159 preterm children at 3.5 years of age. **(A)** Clustering based on the genus taxonomic profile. Biplot arrows indicate the top four genera that drive samples to different locations on the plot. **(B)** The boxplots represent the relative abundance of the top 10 genera distributed among the P_ and B_type enterotypes. The boxplots show the smallest and largest values, 25 and 75% quartiles, the median, and outliers. **(C)** Boxplot of alpha diversity assessed by Chao1 estimate based on the genus taxonomic profile. **(D)** Inter-individual dissimilarity of the genus community based on the Bray–Curtis distance and represented by the heat map and boxplot. Each cell in the heat map, ordered according to B_ and P_type enterotypes, represents the dissimilarity level as per the color scale beside the box plot (*p < 0.05, ****p < 0.0001).

At 3.5 years of age, the gut microbiota of the 159 preterm children was characterized by the presence of six different phyla as follows: *Actinobacteriota*, *Bacteroidota*, *Desulfobacterota*, *Firmicuteota*, *Fusobacteriota*, and *Proteobacteriota*, with *Bacteroidota* and *Firmicuteota* being the most abundant phyla (Supplementary Figure 5). Concerning the stratification of the gut microbiota, it is optimally separated into two distinct enterotypes (Figure 2A). Clustering based on the genus taxonomic level showed that *Prevotella* had the strongest positive correlation and *Bacteroides* had the strongest negative correlation with the PCoA 1 dimension (Figure 2A). The enterotypes were enriched in either *Prevotella* (P_type) (*n* = 31) or *Bacteroides* (B_type) (*n* = 128) (Figure 2B). Interestingly, *Prevotella* was depleted in most children with B_type stools, with a median abundance of 0.02% (IQR, 0.02–0.06%), whereas *Bacteroides* remained the second most abundant genus in children with P_type stools, with a median abundance of 14% (IQR, 6–19%). At the OTU level, the B_type enterotype was characterized by the dominance of OTU_3_*B. vulgatus*, and the P_type enterotype was characterized by the dominance of OTU_30_*P. copri* (Supplementary Figure 6).

When comparing the microbiota of both P_type and B_type enterotypes according to alpha and beta diversities, the P_type enterotype showed greater richness (Chao1 index, *p* = 0.031, genus level) (Figure 2C). At the OTU level, no difference in richness was observed between the two enterotypes (Supplementary Figures 6A,C).

The Bray–Curtis distance pairwise dissimilarities between P_type and B_type enterotypes based on the genus taxonomic profile showed that B_type and P_type children shared a similar bacterial community within each enterotype (Figure 2D). The dissimilarity, indicated by the heatmap color and boxplot of pairwise dissimilarities, was significantly higher in the P_type enterotype than in the B_type enterotype, suggesting that the P_type community was more heterogeneous among children (Figure 2D). In contrast, at the OTU level, the P_type community was less heterogeneous and was characterized by lower pairwise dissimilarity distances (Supplementary Figure 6).

### Preterm and Full-Term Children Gut Microbiota Comparison at 3.5 Years of Age

**FIGURE 3:**
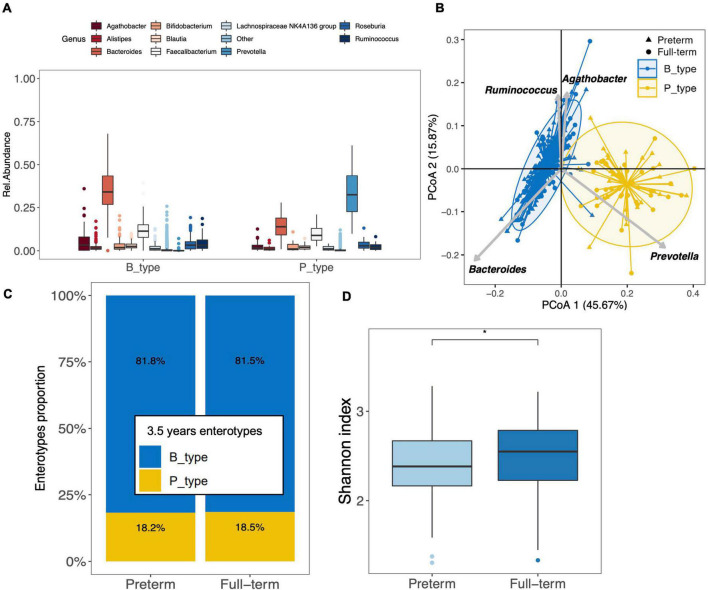
Clustering of the gut microbiota of preterm (*n* = 159) and full-term (*n* = 200) children at 3.5 years of age. **(A)** The boxplots represent the relative abundance of the top 10 genera distributed between the B_ and P_types among the full-term children. The boxplots show the smallest and largest values, 25 and 75% quartiles, the median, and outliers. **(B)** Gut microbiota clustering of the 159 preterm and 200 full-term children at 3.5 years of age based on the genus taxonomic profile. Biplot arrows indicate the top four genera that drive samples to different locations on the plot. **(C)** Distribution of enterotypes among full-term and preterm children. The distribution is expressed as the proportion of each enterotype among each group of children. **(D)** Boxplot of alpha diversity assessed by Shannon index based on the genus taxonomic profile between preterm and full-term children (*p < 0.05). The boxplots show the smallest and largest values, 25 and 75% quartiles, the median, and outliers.

To investigate the differences between the gut microbiota of preterm and full-term children at 3.5 years of age, we included 200 full-term children (ELFE cohort). Full-term children were significantly different in terms of gestational age, delivery mode, birth weight, the rate of mothers born outside of France, and the level of mother’s education (Supplementary Table 2). *Firmicuteota* and *Bacteroidota* were the main phyla identified in the gut microbiota of full-term children. Moreover, the top 10 genera found in preterm children (except for the genus *Blautia*) also characterized the gut microbiota of full-term children (Figure 3A). Enterotyping at the genus taxonomic level showed that the gut microbiota in full-term and preterm children was optimally clustered into two discrete B_type (*n* = 293) and P_type (*n* = 66) enterotypes, with the two largest predictors being *Bacteroides* and *Prevotella*, respectively (Figure 3B). Interestingly, full-term children showed the same patterns as those seen in preterm children, where *Prevotella* was depleted from the majority of B_type children [median abundance of 0.04% (IQR, 0.02–0.08%)], whereas *Bacteroides* remained the second most abundant genus in P_type children (median abundance of 14% [IQR, 9–19%]) (Figure 3A). No differences in enterotype proportion distribution were observed between preterm and full-term children (*p* = 1.000; Figure 3C).

Analysis of alpha diversity showed that the gut microbiota of preterm children was characterized by a significantly lower Shannon diversity index than that of full-term children at the genus (*p* = 0.012) (Figure 3D) and OTU levels (*p* = 0.037). Overall, there were no differences in richness assessed using the Chao1 index at both the genus and OTU levels.

Using both Bray–Curtis and Unifrac distances at the genus level, the PERMANOVA suggested differences in the microbial community between preterm and full-term children (*p* = 0.012 and *p* = 0.006, FDR-corrected, respectively, Supplementary Figure 7).

### Preterm Gut Microbiota Comparison at 1 Month and 3.5 Years of Age

**FIGURE 4:**
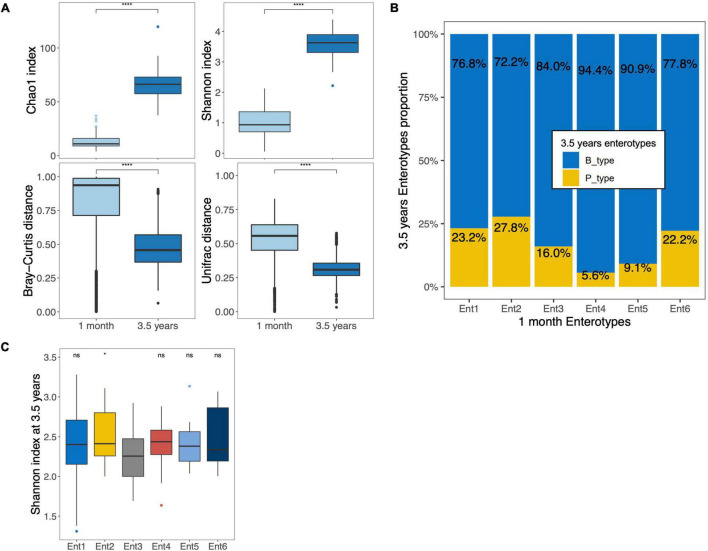
Comparison of the gut microbiota of the same preterm children at 1 month and 3.5 years of age. **(A)** Boxplots of alpha (top) and beta diversities (bottom) based on both Chao1 and Shannon index and both Bray–Curtis and Unifrac dissimilarity distances, respectively, at genus taxonomic level in the 141 preterm children with sequencing data at two time points. **(B)** Distribution of B_ and P_type enterotypes among the six enterotypes at 1 month in 159 preterm children with a fecal sample at two time points. The values are expressed as proportions of each enterotype at 3.5 years of age among each enterotype at 1 month. **(C)** Boxplots of Shannon diversity based on genus level at 3.5 years of age according to enterotype at 1 month (*p < 0.05, ns = *p* > 0.05; Reference group = Ent3, Ent, Enterotype). The boxplots show the smallest and largest values, 25 and 75% quartiles, the median, and outliers. ****p < 0.0001.

Figures 1B, 2B present the top 10 abundant genera at 1 month and 3.5 years of age, respectively, showing that the major genera present in the gut microbiota of preterm children changed between the two ages. In addition to *Bifidobacterium* and *Bacteroides*, the major genera initially found at 1 month (*Enterobacter*, *Clostridium sensu stricto*, *Escherichia*, *Enterococcus*, and *Staphylococcus*) were replaced by other genera (*Agathobacter*, *Faecalibacterium*, *Roseburia*, *Prevotella*, and *Ruminococcus*). Except for *Bifidobacterium* and *Prevotella*, all genera cited above were significantly differentially abundant between the two time points (Supplementary Table 3). Based on the genus composition, the alpha diversity (Chao1 richness and Shannon index) of the gut microbiota significantly increased between both ages (Figure 4A). Pairwise dissimilarities based on genus composition within each age group showed that the gut microbiota of children at 1 month of age harbored higher beta diversity (Bray–Curtis and Unifrac) than at 3.5 years of age (*p* < 0.0001 for both; Figure 4A). Investigation of the relationship between gut microbiota at 1 month and 3.5 years of age in preterm children showed the absence of correlations of diversities between the two age groups either according to alpha diversity (*rho* = −0.054, *p* = 0.521 [Chao1 index]; *rho* = 0.063, *p* = 0.458 [Shannon index]) or beta diversity (*r* = 0.008, *p* = 0.410 [Bray–Curtis]; *r* = 0.030, *p* = 0.176 [Unifrac]). Comparison of the proportions between the two 3.5-year and the six 1-month enterotypes showed that enterotypes 3, 4, and 5 tended to harbor the smallest proportion of P_type, but overall, the association was not significant (Fisher’s exact test, *p* = 0.490) (Figure 4B). Compared to the reference enterotype 3, the gut microbiota of children originally clustered into enterotype 2 presented a higher alpha diversity at 3.5 years of age, but no overall differences were observed (Shannon index, *p* = 0.355; Figure 4C). No overall differences were noted at the OTU level (*p* = 0.173).

### Preterm Gut Microbiota at 3.5 Years of Age and Neonatal Factor Relations

Since we found that enterotypes at 3.5 years of age explained most of the variance in the gut microbiota (PERMANOVA analysis; Supplementary Table 4), we further explored the association between neonatal characteristics and enterotypes. Higher birth weight was significantly associated with the P_type enterotype in the univariate analysis (Table 1). In the multivariate model adjusted for maternal age, education level, and country of birth, we included covariates known to influence early gut microbiota development, such as gestational age, birth mode, birth weight, sex, antibiotics, breastfeeding, skin-to-skin contact, and NICU strategies. The results showed that birth weight was no longer associated with enterotypes, and no other neonatal factors were associated with enterotypes (Supplementary Table 5). Concerning the NICU strategies, we observed a tendency between the practice of a low volume of enteral nutrition and an increased probability of belonging to the P_type enterotype (OR: 5.3, 95% confidence interval [0.9–30.8]) (Supplementary Table 5).

**TABLE 1:** Univariate association between preterm children enterotypes at 3.5 years of age, neonatal characteristics, and NICU strategies.

Variables	Enterotype		
	B_type (n = 128)	P_type (n = 31)	*P*-value
**Gestational age (weeks)**			
Mean (SD)	28.7 (1.96)	29.3 (1.89)	0.117
**Maternal age (years)**			
<25	6 (4.7%)	3 (9.7%)	0.354
25–35	85 (66.4%)	23 (74.2%)	
≥35	32 (25.0%)	5 (16.1%)	
Missing	5 (3.9%)	0 (0%)	
**Country of birth of the mother**			
France	112 (87.5%)	26 (83.9%)	0.810
Other	16 (12.5%)	5 (16.1%)	
**Maternal level of education**			
<High school	24 (18.8%)	11 (35.5%)	**0.011**
High school	19 (14.8%)	8 (25.8%)	
High school diploma +1 +2	30 (23.4%)	7 (22.6%)	
>High school diploma +3	55 (43.0%)	4 (12.9%)	
Missing	0 (0%)	1 (3.2%)	
**Neonatal factors**			
Male sex	66 (51.6%)	20 (64.5%)	0.272
Birth weight (g), Mean (SD)	1130 (335)	1280 (317)	**0.021**
Birth weight (Z-score), Mean (SD)*	−0.913 (1.38)	−0.483 (1.36)	0.123
C-section delivery mode	82 (64.1%)	16 (51.6%)	0.283
**Practice of skin-to-skin contact during the first week of life**			
Started between 0 and 3 days	29 (22.7%)	8 (25.8%)	0.359
Started between 4 and 7 days	39 (30.5%)	13 (41.9%)	
Not practiced	53 (41.4%)	9 (29.0%)	
Missing	7 (5.5%)	1 (3.2%)	
Antibiotic therapy during neonatal period**	104 (81.3%)	28 (90.3%)	1.000
Missing	11 (8.6%)	0 (0%)	
Did the child receive human milk during the neonatal period**	109 (85.2%)	25 (80.6%)	0.796
Missing	3 (2.3%)	1 (3.2%)	
NICU strategy***			
Direct breastfeeding during the first week	11 (8.6%)	2 (6.5%)	0.980
Skin-to-skin contact during the first week	74 (57.8%)	21 (67.7%)	0.419
Longer duration of primary antibiotic therapy	38 (29.7%)	13 (41.9%)	0.273
Longer duration of secondary antibiotic therapy	60 (46.9%)	17 (54.8%)	0.551
Sedation during the first week	81 (63.3%)	20 (64.5%)	1.000
No intubation or extubation at day 1	46 (35.9%)	14 (45.2%)	0.457
Low volume of enteral nutrition at day 7	35 (27.3%)	14 (45.2%)	0.087

*Complete case analysis. Data are presented as number of events (percentages). Missing data are noted if any (B_type, enterotype enriched in Bacteroides; P_type, enterotype enriched in Prevotella).*

*NICU, neonatal intensive care unit.*

*Score based on Olsen curves.

**Neonatal period is defined as the first 28 days of life.

***Favorable strategy: the observed percentage was greater than the expected percentage of newborns receiving the treatment or practice.

*P-values marked with bold indicate statistically significant p-values (p < 0.05).*

## Discussion

This study showed that the gut microbiota of preterm and full-term children at 3.5 years of age is characterized by two enterotypes dominated by either *Bacteroides* or *Prevotella*. Interestingly, we found that prematurity still imprints the gut microbiota of children, as the microbiota of preterm children showed lower diversity and different community composition than that seen in full-term children’s microbiota. Additionally, the gut microbiota at 3.5 years of age was not related to that at 1 month in preterm children.

We confirmed that the gut microbiota of preterm neonates at 1 month of age is highly variable and clustered into six distinct enterotypes. Preterm neonate enterotypes were driven by a specific genus (i.e., *Enterobacter*, *Clostridium sensu stricto 1*, *Escherichia*, *Enterococcus*, and *Staphylococcus*), except for the sixth enterotype, which was characterized by a low bacterial load, leading to the absence of DNA amplification. These findings are in agreement with those of previous studies indicating that the early intestinal microbiota composition in preterm neonates is characterized by a strong dominance of only a few taxa (Stewart et al., 2016; Korpela et al., 2018). In a previous study, one of four genera, *Bifidobacterium*, *Enterobacter*, *Staphylococcus*, or *Enterococcus*, represented > 50% of the reads in a given sample (Korpela et al., 2018). The authors associated *Staphylococcus* and *Enterococcus* dominance with younger postmenstrual age. In our cohort, *Staphylococcus*- and *Enterococcus-*driven enterotypes were also characterized by a lower gestational age (Supplementary Figure 4). Additionally, we found a dominance of *Clostridium sensu stricto 1* and *Escherichia*, and an absence of a *Bifidobacterium-*driven enterotype, even though *Bifidobacterium* was among the top 10 dominant genera at 1 month. Korpela et al. (2018) reported an association between postnatal age and *Bifidobacterium* colonization. As their study was conducted with stool samples collected up to 60 days after birth, the lack of a *Bifidobacterium*-driven enterotype in our study could be explained by the fact that in our cohort, stool samples were collected at a median age of 23 days (IQR, 22–26 days), which is too early for this genus to reach a state of dominance. In a study by Stewart et al. (2016), the gut microbiota of preterm neonates, examined using stool samples collected from birth to 100 days of life, was grouped into six clusters characterized by the dominance of *Klebsiella*, *Staphylococcus*, *Enterococcus*, *Escherichia*, and *Bifidobacterium*, or dominance of both *Klebsiella* and *Enterococcus*.

The present study showed that alpha diversity of the gut microbiota in preterm children increases with age and that the gut microbiota community is significantly different between 1 month and 3.5 years of age. The latter is characterized by two discrete enterotypes driven by two dominant genera, *Bacteroides* and *Prevotella*. Previous studies monitoring the gut microbiota of preterm children between the NICU stay and 2 years of age also showed a significant increase in alpha diversity with age (Gómez et al., 2017; Yee et al., 2019) and reported differential beta-diversity structures between NICU stay and 4 years of age (Yee et al., 2019). We observed greater variability in the diversity of preterm children’s gut microbiota at 1 month than at 3.5 years; this finding is in accordance with previous reports (Barrett et al., 2013; Stewart et al., 2016; Korpela et al., 2018). This indicates that preterm neonates’ early gut microbiota has greater inter-individual variability and lower stability but evolves and stabilizes over time. Furthermore, between 1 month and 3.5 years, 68 genera were significantly differentially abundant (25 negatively and 43 positively associated with the gut microbiota at 3.5 years). Indeed, in addition to *Bifidobacterium* and *Bacteroides*, the major genera shifted from organisms previously found to be abundant in preterm neonates during the NICU stay to common organisms found in adults, such as *Faecalibacterium*, *Roseburia*, and *Ruminococcus* (Roswall et al., 2021). Altogether, these data support the fact that the intestinal microbiota of children born preterm evolves toward a richer and more diverse adult-like microbiota over time.

Analyses of correlations between preterm children’s gut microbiota diversity during the NICU stay and early childhood showed no significant relationship. Children with higher alpha diversities at 1 month of age did not harbor higher alpha diversities at 3.5 years of age. Based on beta diversity, we found no correlation when examining community composition among close-together (i.e., lower dissimilarity distance) preterm children at 1 month and at 3.5 years of age, demonstrating that close-together newborns do not conserve their proximity over time. Similarly, there was no association between preterm neonate enterotypes at 1 month and 3.5 years of age, suggesting that belonging to one of the six enterotypes at 1 month did not predispose to harbor a certain type of enterotype at 3.5 years. If clustering methods are sensitive to sample size (Koren et al., 2013; Henry et al., 2015; Preud’homme et al., 2021), in the present study, we demonstrated good reproducibility of the clustering at 1 month based on our population of 141 EPIFLORE preterm neonates (i.e., 159 neonates excluding the 18 preterm neonates belonging to enterotype 6) compared to the previous clustering performed on 484 EPIFLORE preterm neonates (Rozé et al., 2020). Because we identified the same clustering patterns based on smaller sample size, we can assume that the shift from six to two enterotypes that we observed is not an artifact of the smaller sample size of the present study.

In the present study, the analysis between preterm and full-term children at 3.5 years of age showed that both populations shared similar microbiota profiles characterized by *Bacteroides-* and *Prevotella-*driven enterotypes. This clustering is consistent with the gut microbiota studies among full-term children (Bergström et al., 2014; Nakayama et al., 2015; Méndez-Salazar et al., 2018; Xiao et al., 2021) and adults (Roager et al., 2014; Levy et al., 2020). A comparison of the microbiota community complexities at 3.5 years of age indicated that preterm and full-term children shared a similar richness composition, but preterm children had a lower alpha diversity and a different beta diversity (Supplementary Figure 7). It is known that the early gut microbiota differs between full-term and preterm children because of differences in exposure to prenatal and postnatal determinants (Groer et al., 2014; Tauchi et al., 2019; Aguilar-Lopez et al., 2021). Nonetheless, little is known about how early differences in gut colonization and establishment evolve as preterm neonates grow older. Differences in gut microbiota diversity between preterm and full-term children are observed within the first 6 months of life and are either still present (Vandenplas et al., 2020) or disappear (Dahl et al., 2018; Yap et al., 2021) at 12 months of age. Recently, the transition of enterotypes shaping the gut microbiota of children at an early age revealed that gut microbiota maturity of the preterm children was delayed up to 12 months compared to that of full-term children (Xiao et al., 2021). The present study indicates that at 3.5 years of age, the gut microbiota of the preterm children differs in terms of composition and is less diverse than that of full-term children’s gut microbiota, suggesting that the transition recovery of the initially delayed gut microbiota colonization in preterm children still continues at 3.5 years of age. Our results are in accordance with those of Fouhy et al. (2019), who reported that gestational age imprints gut microbiota up to 4 years of age. Over the course of 3.5 years, we observed the evolution of preterm children’s gut microbiota toward an enterotype-like gut microbiota similar to those found in full-term children. Because there is currently a lack of data on the development of preterm children’s gut microbiota during early childhood and its impact on later health outcomes, the present study provides another insight into our understanding of the impact of gestational age on gut microbiota development up to 3.5 years of age.

The neonatal factors, such as gestational age, delivery mode, breastfeeding, early antibiotic therapy medication, skin-to-skin practice, and NICU practices, are known to influence early gut microbiota colonization (Rodríguez et al., 2015; Martin et al., 2016; Tamburini et al., 2016; Kumbhare et al., 2019; Rozé et al., 2020; Vandenplas et al., 2020). However, little is known about the long-term impact of these factors on the gut microbiota. Therefore, we investigated the potential effects of these factors on the gut microbiota of preterm children aged 3.5 years. We found no effects on the gut microbiota composition or any significant associations with the enterotype prevalence in preterm children at 3.5 years of age, indicating that these factors may not persist up to this age. Nonetheless, we observed a tendency between a low enteral volume at 7 days and an increased probability of belonging to the *Prevotella-*driven enterotype; the odds ratio indicates a strong association, but the wide 95% confidence interval indicates poor precision, which may be due to the limited sample size. Previously, the EPIFLORE study reported that gestational age and birth mode were associated with enterotypes at 1 month of age. Furthermore, no assisted ventilation on day 1 was associated with a decreased risk of belonging to enterotypes 5 or 6, while sedation and low volume of enteral nutrition were associated with an increased risk (Rozé et al., 2020), [enterotypes 5 and 6 were the less mature enterotypes characterized by a smaller gestational age (Supplementary Figure 4)]. If NICU practices are associated with preterm neonates’ gut microbiota during the NICU stay, some of these practices may remain weakly associated with the microbiota 3.5 years later.

### Study Limitations and Strengths

The use of the 16S rRNA gene sequencing approach limits this study to the description of bacterial composition. Analysis using shotgun metagenomics would allow greater sequencing depth, thus providing additional information regarding microbiome functional profiles and meaning at the species level of the microbiome that can differ between preterm and full-term populations, despite similar bacterial composition. Microbiota analysis was only performed for two times, that is, at 1 month and 3.5 years of age, which does not take into account possible dynamic changes in microbiota between these two time points. Moreover, we studied the influence of prenatal factors on the gut microbiota at 3.5 years in a preterm population, but we did not take into account more contemporary factors occurring during childhood, such as the use of antibiotics or dietary habits that might influence the gut microbiota at 3.5 years. Finally, the number of children included may represent a limitation to definitely conclude about the association between the gut microbiota at 3.5 years of age and neonatal factors, but a tendency was observed, indicating a potential lack of statistical power.

In terms of major strengths, the present study is rare in the number of preterm children included within a multi-year follow-up and was performed on a large nationwide multicentric cohort, providing an accurate description of the neonatal characteristics of preterm children and characterization of the NICU strategies during hospitalization. Therefore, it allows a confident description of the preterm gut microbiota at both 1 month and 3.5 years of age, when the children’s gut microbiota is supposed to mature toward an adult-like microbiota.

## Conclusion

This study demonstrates that during the NICU stay, the gut microbiota of preterm neonates is characterized by high inter-individual variation and low stability shaped by the NICU environment. However, NICU-shaped gut microbiota was not predictive of microbial community composition in the same child at 3.5 years of age. At 3.5 years of age, preterm children share gut microbiota similar to that of children born full-term and correspond to a mature adult-like microbiota in terms of enterotypes. However, the gut microbiota of preterm children was characterized by a lower Shannon diversity and different overall microbial composition compared to that seen in the gut microbiota of full-term children, indicating that prematurity still imprints the gut microbiota up to 3.5 years of age. These results must be replicated in other large-scale longitudinal cohorts to confirm the relationship between gestational age and other perinatal factors and the gut microbiota during early childhood.

## Data Availability Statement

Personal data of children from the ELFE and EPIPAGE2 cohorts cannot be made publicly available for ethical reasons. They are available upon reasonable request from the authors under data-security conditions. The 16S rRNA gene reads are publicly available from the National Center for Biotechnology Information (nih.gov) Sequence Read Archive (SRA) under the Bioproject accession number: PRJNA798897, https://www.ncbi.nlm.nih.gov/bioproject/PRJNA798897.

## Ethics Statement

This study was approved by the National Data Protection Authority (Commission Nationale de l’Informatique et des Libertes [CNIL]), by the national advisory committee on information processing in health research (Comité Consultatif sur le Traitement de l’Information en matière de Recherche dans le domaine de la Santé [CCTIRS]) and by the Committee for the Protection of People Participating in Biomedical Research (Comité de Protection des Personnes [CPP]). Recruitment and data collection occurred only after the families had received information and agreed to participate in these cohorts with informed consent.

## The EPIFLORE Study Group

The members of the group include: M-JB, JA, Clotilde Rousseau, and JD (Federation of University Hospital, PREMA, UMR-S 1139, Faculty of Pharmacy, INSERM and Paris Descartes University); PL, Joel Dore, PhD, Ziad Al Nabhani, PhD, Karine Le Roux, and Celine Monot (INRA, UMR 1319 MICALIS); J-CR (Department of Neonatal Medicine, Nantes University Hospital); P-YA, Laetitia MartinMarchand, and Melanie Durox (INSERM, U1153, Obstetrical, Perinatal and Pediatric Epidemiology Team, Epidemiology and Biostatistics Sorbonne); Alexandre Lapillonne, MD, PhD (Department of Neonatal Medicine, Assistance Publique Hopitaux de Paris, Necker Enfants Malades Hospital); Jean-Charles Picaud, MD, PhD (Department of Neonatal Medicine, Hopital de la Croix-Rousse, Hospices Civils de Lyon); Farid Boudred, MD, PhD (Department of Neonatology, Faculte de Medecine, Aix-Marseille Université); Delphine Mitanchez, MD, PhD (Division of Neonatology, Department of Perinatology, Armand Trousseau Hospital); Valerie Biran (Department of Neonatalogy, Université Paris 7, Robert-Debre Hospital, Assistance Publique Hopitaux de Paris); Laurent Storme, MD, PhD (Department of Neonatal Medicine, Lille University Hospital); Olivier Claris, MD, PhD (Mothers and Children Hospital, Hospices Civils de Lyon); Gilles Cambonie, MD, PhD (Department of Neonatal Medicine, Montpellier University Hospital); Cyril Flamant, MD, PhD (Department of Neonatal Medicine, Nantes University Hospital); Anne Sauret, MD (Department of Neonatal Medicine, Rennes University Hospital); Odile Dicky, MD (Department of Neonatal Medicine, Toulouse University Hospital); Geraldine Favrais, MD, PhD (Department of Neonatalogy, Tours University Hospital); Jean-Michel Hascoet, MD, PhD (Department of Neonatal Medicine, Nancy University Hospital); Geraldine Gascoin (Department of Neonatal Medicine, Angers University Hospital); Gerard Thiriez, MD, PhD (Department of Pediatrics, Besancon University Hospital); Luc Desfrere, MD, PhD (Department of Neonatal Medicine, Louis Mourier Hospital, Assistance Publique Hopitaux de Paris); Xavier Durrmeyer, MD, PhD (Department of Neonatal Medicine, Centre Hospitalier Intercommunal); and Clement Chollat, MD (Department of Neonatal Medicine, Cochin University Hospital).

## Author Contributions

J-CR, M-JB, M-AC, P-YA, and JA conceived and designed the study. M-JB, PL, GT, JD, and J-CR generated the data. JD and GT analyzed the data. GT drafted the manuscript. M-JB, J-CR, PL, JD, P-YA, M-AC, and JA critically revised the manuscript. JA, M-JB, and M-AC supervised the study. All authors approved the submitted version.

## Conflict of Interest

The authors declare that the research was conducted in the absence of any commercial or financial relationships that could be construed as a potential conflict of interest.

## Publisher’s Note

All claims expressed in this article are solely those of the authors and do not necessarily represent those of their affiliated organizations, or those of the publisher, the editors and the reviewers. Any product that may be evaluated in this article, or claim that may be made by its manufacturer, is not guaranteed or endorsed by the publisher.
