# Supplementary material for: Very Preterm Children Gut Microbiota Comparison at the Neonatal Period of 1 Month and 3.5 Years of Life
Source: Front Microbiol. 2022 Jul 22;13:919317. doi: 10.3389/fmicb.2022.919317 (PMC9354809; doi:10.3389/fmicb.2022.919317)
Supplement: Supplementary file 1 [file Table_1.DOCX]

**Table S1.** Characteristics comparison of preterm children with a fecal sample at 1 month and 3.5 years of age with those of preterm children with only one or no fecal sample in the EPIFLORE cohort.

| **Variables** | **Preterm children with fecal collect at 1 month and 3.5 years of age**  **(n=159)** | **Preterm children with 1 or no fecal sample (n=570)** | **P value** |
| --- | --- | --- | --- |
| **Gestational age (Weeks)** |  |  |  |
| Mean (SD) | 28.8 (1.96) | 28.9 (2.15) | 0.732 |
| **Maternal age (year)** |  |  |  |
| <25 | 9 (5.7%) | 114 (20.0%) | <0.001 |
| [25-35[ | 108 (67.9%) | 305 (53.5%) |  |
| >=35 | 37 (23.3%) | 131 (23.0%) |  |
| Missing | 5 (3.1%) | 20 (3.5%) |  |
| **Country of birth of the mother** |  |  |  |
| France | 138 (86.8%) | 401 (70.4%) | <0.001 |
| Other | 21 (13.2%) | 164 (28.8%) |  |
| Missing | 0 (0%) | 5 (0.9%) |  |
| **Maternal level of education** |  |  |  |
| < High school | 35 (22.0%) | 155 (27.2%) | 0.0149 |
| High school | 27 (17.0%) | 111 (19.5%) |  |
| High school diploma +1 +2 | 37 (23.3%) | 101 (17.7%) |  |
| >High school diploma +3 | 59 (37.1%) | 131 (23.0%) |  |
| Missing | 1 (0.6%) | 72 (12.6%) |  |
| **Neonatal factors** |  |  |  |
| **Sex** |  |  |  |
| Boy | 86 (54.1%) | 301 (52.8%) | 0.844 |
| Girl | 73 (45.9%) | 269 (47.2%) |  |
| **Birth weight (g)** |  |  |  |
| Mean (SD) | 1160 (336) | 1150 (343) | 0.857 |
| **Delivery mode** |  |  |  |
| Vaginal | 61 (38.4%) | 208 (36.5%) | 0.768 |
| C-section | 98 (61.6%) | 359 (63.0%) |  |
| Missing | 0 (0%) | 3 (0.5%) |  |
| **Practice of skin-to-skin contact during the first week of life** |  |  |  |
| Started between 0-3 days | 37 (23.3%) | 126 (22.1%) | 0.961 |
| Started between 4-7 days | 52 (32.7%) | 173 (30.4%) |  |
| No practiced | 62 (39.0%) | 219 (38.4%) |  |
| Missing | 8 (5.0%) | 52 (9.1%) |  |
| **Antibiotherapy during neonatal period*** |  |  |  |
| No | 16 (10.1%) | 40 (7.0%) | 0.435 |
| Yes | 132 (83.0%) | 443 (77.7%) |  |
| Missing | 11 (6.9%) | 87 (15.3%) |  |
| **Did the child receive human milk during neonatal period*** |  |  |  |
| No | 21 (13.2%) | 59 (10.4%) | 0.862 |
| Yes | 134 (84.3%) | 346 (60.7%) |  |
| Missing | 4 (2.5%) | 165 (28.9%) |  |

Complete cases analysis. Data are number of events (percentages).

*Neonatal period is defined as the 28 first days of life

**Table S2.** Population characteristics of the ELFE full-term and EPIFLORE preterm children included in the present study.

|  | **Full-term** | **Preterm** | **P-value** |
| --- | --- | --- | --- |
|  | **(N=200)** | **(N=159)** |  |
| **Gestational age (Weeks)** |  |  |  |
| Mean (SD) | 39.8 (1.03) | 28.8 (1.96) | <0.001 |
| **Sex** |  |  |  |
| Boys | 121 (60.5%) | 86 (54.1%) | 0.265 |
| Girls | 79 (39.5%) | 73 (45.9%) |  |
| **Delivery mode** |  |  |  |
| Vaginal | 157 (78.5%) | 61 (38.4%) | <0.001 |
| Cesarean | 39 (19.5%) | 98 (61.6%) |  |
| Missing | 4 (2.0%) | 0 (0%) |  |
| **Birth weight (Grams)** |  |  |  |
| Mean (SD) | 3400 (421) | 1160 (336) | <0.001 |
| Missing | 1 (0.5%) | 0 (0%) |  |
| **Maternal age (Years)** |  |  |  |
| <25 | 6 (3.0%) | 9 (5.7%) | 0.209 |
| [25-35[ | 155 (77.5%) | 108 (67.9%) |  |
| ≥35 | 39 (19.5%) | 37 (23.3%) |  |
| Missing | 0 (0%) | 5 (3.1%) |  |
| **Mother born in France** |  |  |  |
| Yes | 190 (95.0%) | 138 (86.8%) | 0.0104 |
| No | 10 (5.0%) | 21 (13.2%) |  |
| **Maternal level of education** |  |  |  |
| < High School | 4 (2.0%) | 35 (22.0%) | <0.001 |
| High school | 43 (21.5%) | 27 (17.0%) |  |
| High school diploma +1 +2 | 41 (20.5%) | 37 (23.3%) |  |
| > High school diploma +3 | 110 (55.0%) | 59 (37.1%) |  |
| Missing | 2 (1.0%) | 1 (0.6%) |  |
| **Maternal BMI before pregnancy** |  |  |  |
| Underweight | 13 (6.5%) | 7 (4.4%) | 0.483 |
| Normal | 135 (67.5%) | 111 (69.8%) |  |
| Overweight | 40 (20.0%) | 22 (13.8%) |  |
| Obese | 12 (6.0%) | 10 (6.3%) |  |
| Missing | 0 (0%) | 9 (5.7%) |  |
| **Parity** |  |  |  |
| Primipara | 89 (44.5%) | 71 (44.7%) | 1 |
| Multipara | 109 (54.5%) | 88 (55.3%) |  |
| Missing | 2 (1.0%) | 0 (0%) |  |

Data are number of events (percentages).

**Table S3.** Differential abundance testing analysis between samples at 1 month (n=141) and 3.5 years of age (n=159) in the preterm population

Direction: - = less abundant taxa in the category compared to the reference, + = more abundant taxa in the category compared to the reference. ANCOM-BC: beta= coefficient obtained from the ANCOM-BC log linear (natural log) model (log-transformed change in abundance), se = standard error of the beta, W = test statistic (beta/se). ALDEx2: effect= per-feature effect size, overlap =per-feature proportion of effect size that is 0 or less, diff.btw= per-feature median difference between the two conditions (3.5y vs 1 month), diff.win=p er-feature maximum median difference between Dirichlet instances within conditions.

adj.pvalue = False discovery rate (FDR) adjusted p-value

**Table S5.** Multivariate analysis of the characteristics of the EPIFLORE preterm children and the treatment strategies of the NICU where children were hospitalized at day 7 according to the enterotypes at 3.5 years of age.

|  | **Enterotype P_type vs B_type** | |
| --- | --- | --- |
|  | Odds Ratios† [CI] | p-value |
| **Neonatal factors** |  |  |
| Gestational age (weeks) | 0.86 [0.54 – 1.38] | 0.532 |
| Sex [Girl] | 0.48 [0.16 – 1.48] | 0.203 |
| Birth weight (grams) | 1.00 [1.00 – 1.01] | 0.082 |
| Delivery mode [C-section] | 0.77 [0.27 – 2.21] | 0.627 |
| Practice of skin-to-skin contact during the first week of life [Start between 0-3 days] | 0.56 [0.11 – 2.77] | 0.478 |
| Practice of skin-to-skin contact during the first week of life [Start between 4-7 days] | 1.22 [0.31 – 4.84] | 0.773 |
| Antibiotherapy during neonatal period [Yes] | 1.44 [0.20 – 10.17] | 0.717 |
| Does the child received human milk during neonatal period [Yes] | 1.45 [0.28 – 7.51] | 0.661 |
| **NICU's strategy*** |  |  |
| Direct breastfeeding during the first week [Yes] | 0.91 [0.06 – 12.83] | 0.942 |
| Skin-to-skin contact during the first week [Yes] | 1.29 [0.23 – 7.42] | 0.773 |
| Longer duration of primary antibiotherapy [Yes] | 2.67 [0.49 – 14.44] | 0.254 |
| Longer duration of secondary antibiotherapy [Yes] | 2.14 [0.53 – 8.69] | 0.287 |
| Sedation during the first week [Yes] | 1.81 [0.47 – 6.90] | 0.386 |
| No intubation or extubation at day 1 [Yes] | 0.42 [0.05 – 3.52] | 0.423 |
| Low volume of enteral nutrition at day 7 [Yes] | 5.27 [0.90 – 30.81] | 0.065 |
|  |  |  |

Complete case analysis (n=133). Mixed-effects logistic regression with a random hospital intercept.

†Odds ratio adjusted for mother age, maternal education, and country of birth of the mother.

Abbreviations: NICU, neonatal intensive care unit.

*Favorable strategy, the observed percentage was zero or greater than the expected percentage of infant receiving the treatment or practice.

**Figure S1. EPIFLORE and ELFE cohorts flow chart.** Dates on the lefts of the figure correspond to the year of collection of fecal samples for the two different time points.

**
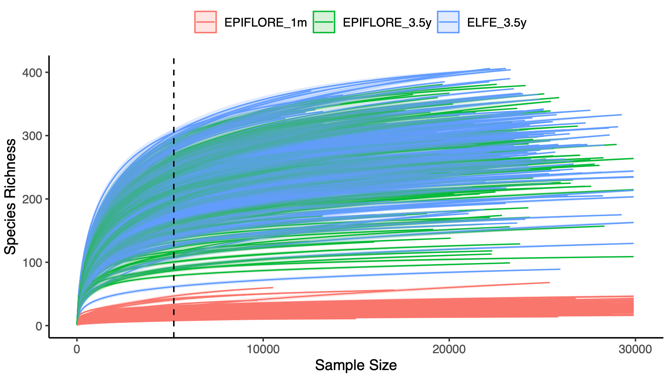
**

**Figure S2. Rarefaction curves of samples colored by sample groups.** Vertical dash line represents the 5201 pb threshold.

**Figure S3. Description of gut microbiota clustering 1 month after birth (n=141) in preterm infants** **based on OTU profiles**. **(A)** The boxplots represent the relative abundance of the top 10 OTUs distributed among the 5 clusters. **(B)** Boxplots of alpha diversity (top) assessed by Chao1 and Shannon index and pairwise beta diversity distance (bottom) assessed by Bray and Unifrac distances between clusters based on OTUs taxonomic profiles. The boxplots show the smallest and largest values, 25% and 75% quartiles, the median and outliers.  (**p* < 0.05, ***p* < 0.01, *****p* < 0.0001, ns=p>0.05, Ref group=Ent3). The sixth cluster, constituted by infants in whom no amplification could be performed owing to a low bacterial load, is not represented by definition.

Compared to enterotype 3, Ent4 and Ent5 showed lower Chao1 richness (p<0.05 and p<0.01 respectively) and no or lower diversity (p<0.05) according to Shannon index respectively.

Ent1 and 2 harbored no differences for both indexes. However, uneven overall alpha diversity according to both Chao1 and Shannon diversity indexes was described (p=0.005 and p=0.008 respectively). Pairwise beta-diversity comparisons showed overall differences across groups according to both Bray-Curtis and Unifrac distance dissimilarities (p<10^-4^). Compared to Ent3, Ent 1, 2 and 4 showed higher Bray-Curtis pairwise dissimilarities among infants belonging to these enterotypes (p< 0.0001 for all) meaning higher inter-individuality variation but lower pairwise dissimilarities with Unifrac distance. No differences were noticed for Ent5.

**Figure S4. Gestational age according to the enterotypes describing the gut microbiota at 1 month after birth in the preterm children (n=159).** The boxplots show the smallest and largest values, 25% and 75% quartiles, the median and outliers.  (**p* < 0.05, ****p* < 0.001, ns=p>0.05, Ref group=Ent3).

**Figure S5. Composition of the gut microbiota of preterm children at 3.5 years of age (n=159).** Abundances are expressed as relative abundance.

**Figure S6. Description of gut microbiota clustering at 3.5 years (n=159) in preterm children based on the OTU profiles. (A)** Boxplots of alpha diversity assessed by Chao1 and Shannon indexes. **(B)** Pairwise beta diversity dissimilarities assessed by Bray-Curtis distance between the two enterotypes The boxplots show the smallest and largest values, 25% and 75% quartiles, the median and outliers (ns=p>0.05). **(C)** The number of OTU was determined in each infant at each sampling depth in 200-read increment. The means and standard deviation of the P_type group (n = 28) and the B-type group (n = 131) are shown in the rarefaction plot. **(D)** The boxplots represent the relative abundance of the top 10 OTUs distributed among the two enterotypes. All analyses are based on OTUs taxonomic profiles.

**Figure S7. Preterm (n=159) and full-term (n=200) principal coordinate analysis (MDS) plots of the Bray-Curtis and Unifrac distances at the genus (A) and OTU (B) taxonomic profiles.** Preterm and full-term born children are represented by different colors.

Concerning community complexities, using both Bray-Curtis and Unifrac distances, the PERMANOVA suggested differences in microbiota community structure between preterm and full-term infants at both genus (R^2^ = 0.010, p = 0.012 and R^2^ = 0.008, p =0.006 respectively; FDR corrected) and OTU taxonomic levels (R^2^ = 0.007, p = 0.009 and R^2^ = 0.008, p =0.006 respectively; FDR corrected).
